# ARBRES: Light-Weight CW/FM SAR Sensors for Small UAVs

**DOI:** 10.3390/s130303204

**Published:** 2013-03-06

**Authors:** Albert Aguasca, Rene Acevo-Herrera, Antoni Broquetas, Jordi J. Mallorqui, Xavier Fabregas

**Affiliations:** Remote Sensing Lab, Department of Signal Theory and Communications, Universitat Politècnica de Catalunya, UPC Campus Nord D3, E-08034 Barcelona, Spain; E-Mails: rene.acevo@gmail.com (R.A.-H.); broquetas@tsc.upc.edu (A.B.); mallorqu@tsc.upc.edu (J.J.M.); fabregas@tsc.upc.edu (X.F.)

**Keywords:** UAV, SAR, CW/FM, MoCo, interferometry

## Abstract

This paper describes a pair of compact CW/FM airborne SAR systems for small UAV-based operation (wingspan of 3.5 m) for low-cost testing of innovative SAR concepts. Two different SAR instruments, using the C and X bands, have been developed in the context of the ARBRES project, each of them achieving a payload weight below 5 Kg and a volume of 13.5 dm^3^ (sensor and controller). Every system has a dual receiving channel which allows operation in interferometric or polarimetric modes. Planar printed array antennas are used in both sensors for easy system integration and better isolation between transmitter and receiver subsystems. First experimental tests on board a 3.2 m wingspan commercial radio-controlled aircraft are presented. The SAR images of a field close to an urban area have been focused using a back-projection algorithm. Using the dual channel capability, a single pass interferogram and Digital Elevation Model (DEM) has been obtained which agrees with the scene topography. A simple Motion Compensation (MoCo) module, based on the information from an Inertial+GPS unit, has been included to compensate platform motion errors with respect to the nominal straight trajectory.

## 1. Introduction

Different geophysical and biophysical parameters can be monitored with Synthetic Aperture Radar (SAR) sensors in applications such as earthquake damage assessment, subsidence mapping, harvest monitoring, deforestation and fire impact assessment, DEM production and oil spill monitoring [1–5]. In contrast to orbital platforms with rigid revisit times, airborne SAR provides the observation flexibility required in time-critical or fast dynamic applications. However the high operational costs and sensor availability limit the application of airborne SAR. Unmanned Aerial Vehicles (UAVs) offer an interesting cost effective alternative for airborne SAR remote sensing. UAVs can be remotely piloted and continuously operated for many hours, the take-off and landing requirements are small, and their maintenance is simpler than manned aircraft. On the other hand energy availability, flight regulations and safety concerns limit the spatial coverage of UAV-SAR missions. For these reasons the development of SAR for UAVs is particularly interesting for low-cost testing of innovative SAR concepts, based on new instrument architectures or requiring access to raw-data databases of different scenarios, which are increasingly difficult to obtain. Representative examples of some UAV-SAR instruments are nanoSAR (ImSAR Co., Springville, UT, USA), microASAR [6] (Artemis, Inc., Hauppauge, NY, USA) and miniSAR (Sandia Nat. Labs, Albuquerque, NM, USA). In the presented case we show that multichannel operation in several bands can be achieved with small UAV platforms, which is interesting for experimental evaluation of SAR interferometric and polarimetric applications.

**Figure 1.**
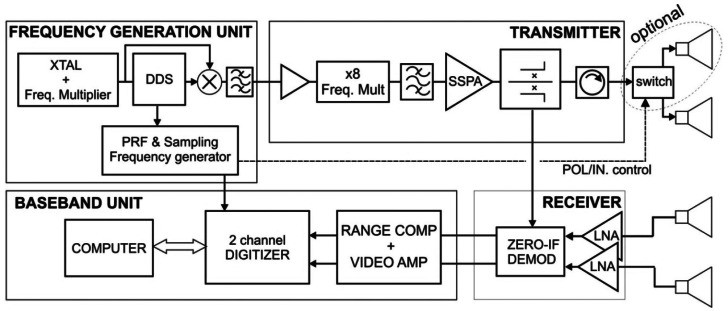
ARBRES-X block diagram. ARBRES-C is similar, with a different frequency multiplier (×4) in the transmitter block.

The design and development of a SAR sensor to be fitted to a UAV platform imposes strong constraints in compactness, weight, power consumption, and robustness. Taking these into account, two experimental short to medium range C and X-band SAR sensors have been developed with single-pass interferometric or polarimetric capabilities. The AiR-Based REmote Sensing (ARBRES) SAR sensors are light-weight and stand-alone operable instruments that work with a Stepped Linear Frequency Modulated Continuous Wave (SLFM-CW) signal. Their radar architecture is based on the previously developed Ground Based-SAR [7,8] with Commercially available Off-The-Shelf (COTS) components in the RF, IF, control and data storage sections (Figure 1).

In this case the emphasis has been placed on weight and volume reduction without loss of the radar performance. This project is affordable for an unsophisticated laboratory with basic microwave instrumentation, because it uses simple RF and microwave circuitry based on microstrip technology.

## 2. System Description

The Universitat Politècnica de Catalunya (UPC) ARBRES SAR project consists of two independent single transmitter/dual receiver channel sensors. They have been designed to operate in the C-band (5.3 GHz, ARBRES-C) and X-band (9.65 GHz, ARBRES-X) using a SLFM-CW signal. The SLFM-CW waveform is generated by a Direct Digital Synthesizer (DDS). The CW operation simplifies notably the system structure with respect to pulsed radars. Both sensors are based on solid-state 1Watt output power transmitters. ARBRES-C works only in polarimetric configuration, since the limited size of the available UAV does not allow enough receiver antennas separation to achieve a useful interferometric baseline. The higher frequency of the X-band SAR allows single-pass Interferometric (InSAR) observations as well as Polarimetric ones (PolSAR). Table 1 summarizes the list of the main specifications of the system. In both instruments, the receiver unit consists of two parallel low-noise chains with a direct I/Q Zero-IF demodulator, where a sample of the transmitted signal is used as local oscillator.

The baseband signal acquisition is performed by a commercial 14 bit, 2 channels, high speed digitizer (up to 65Ms/s per channel and 512 MB memory depth) in PCI format, controlled by a single board computer with a solid state hard disk drive. The two baseband signals are synchronously digitized using trigger and clock references that are coherently generated in the Frequency Generation Unit.

The system incorporates a commercial Inertial Motion Unit (IMU) integrated with a Global Positioning Satellite (GPS) receiver that provides the position and the attitude of the platform (*X-Sens* MTI-G unit, with a 2.5 m position accuracy and an angular dynamic accuracy of 1 deg RMS). The position and attitude information are stored simultaneously, together with the radar raw data for a proper image geocoding.

## 3. Internal Structure

**Figure 2.**
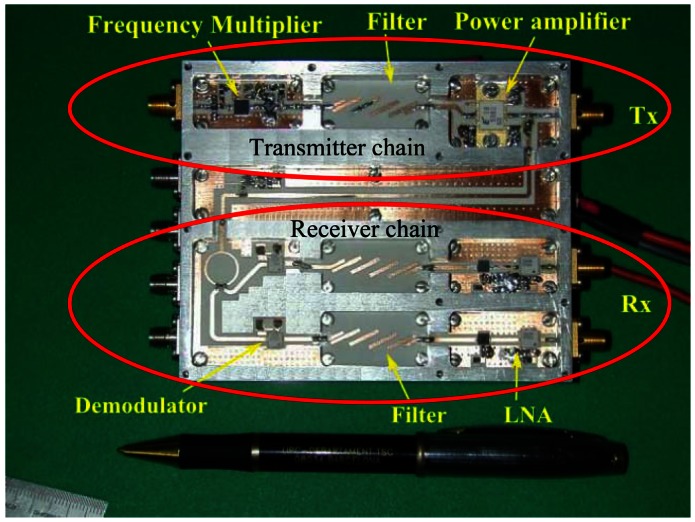
RF front end of ARBRES-X. Different planar circuits are placed in a mechanized aluminium box of 110 × 90 × 30 mm^3^. The bottom of the box includes the biasing network and part of the Base-Band circuitry.

**Figure 3.**
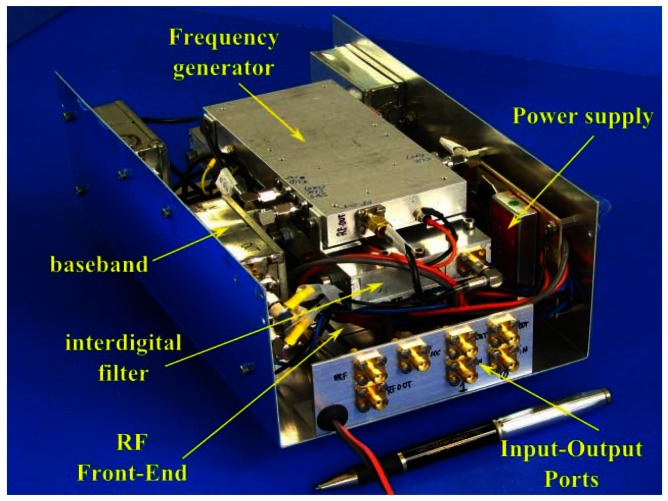
Integrated ARBRES-X System. The box, an aluminium enclosure of 255 × 155 × 90 mm^3^, contains the RF, frequency generation and the baseband unit.

Figure 1 shows the block diagram of the X-band version. The C-band version follows the same structure. Every receiver unit consists of two parallel low-noise chains with a direct I/Q Zero-IF demodulator, where a sample of the transmitted signal is used as local oscillator. In this way the echo signal is directly deramped [7], reducing considerably the bandwidth and storage requirements of data. A picture of the X-band front end is shown in Figure 2, and a picture of the complete instrument is shown in Figure 3.

The frequency generation unit is based on a Direct Digital Synthesizer chipset that generates the SLFM-CW signal in the 200–300 MHz range. The number of frequency steps and the step size have been optimized to reduce undesirable artifacts in the detection process [9]. The DDS signal is upconverted to 1.2 GHz using the same DDS clock signal as Local Oscillator. In a first attempt the system worked with the equivalent high frequency Nyquist harmonic of the DDS [10], but it had to be discarded because its spectral quality. An active MMIC frequency multiplier generates the harmonic to be transmitted and expands the signal bandwidth; this simplifies the transmitter chain structure because no intermediate local oscillators are needed.

**Figure 4.**
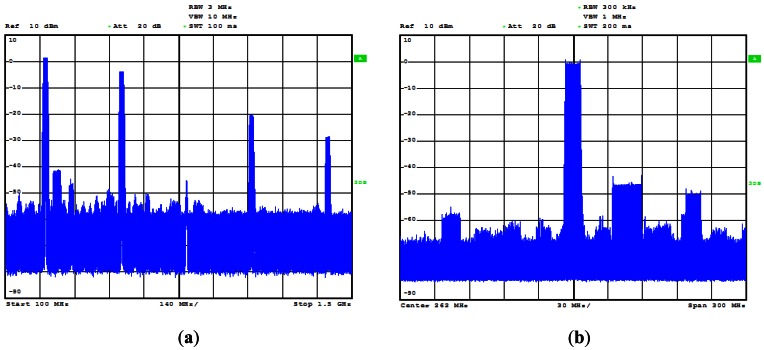
Frequency spectrum of the 262 MHz centered, 12 MHz bandwidth triangular SLFM-CW signal generated by the DDS (before filtering and up-conversion process). (**a**) 1.4 GHz span and (**b**) detailed span of 300 MHz.

The DDS output signal has low phase noise, but affected by spurs (Figure 4) due to the truncation after the phase accumulator in its internal structure. To obtain a spurious-free signal, a highly selective band-pass filter must be inserted after the first up-conversion of the DDS output. Different filter structures and technologies have been tested, including the suspended bar interdigital filters (the best in terms of frequency selectivity but big and heavy) and the RF SAW filters (the smallest and lightest option). A commercial solid-state MMIC power amplifier generates the 1W output signal, that is biased with a low ripple 5V DC/-5V DC power supply.

As usual in CW radar operation, the coupling between transmitter and receiver is critical to avoid saturation of the receiver front end. The isolation requirement has been achieved using separated transmitting and receiving antennas. The adopted DDS can be programmed to generate the linear FM signal following either a Triangular or a Saw-tooth pattern. In our design the Triangular FM is preferable since the Saw-Tooth abrupt frequency transitions causes unwanted impulses in the IF signal.

**Figure 5.**
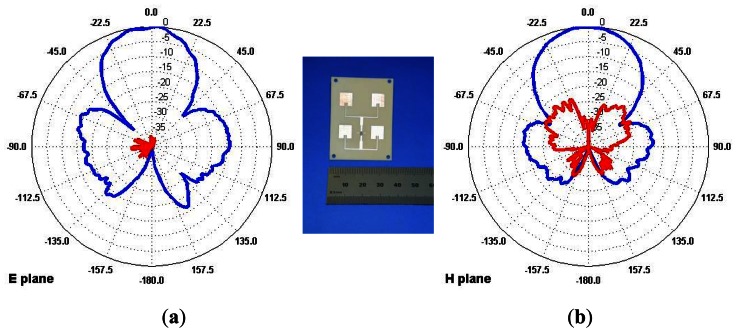
Measured Antenna patterns. The blue lines correspond to the normalized radiation diagram cuts, in dB, in E plane (**a**) and H plane (**b**). The red lines correspond to the Cross Polarization Discrimination factor, in dB, for both planes.

The antenna coupling complicates notably the antenna design and location in a small UAV, in this case, both sensors use pairs of arrays of four microstrip patch radiators (2 × 2, λ/2 spaced, patch array), with a 2-way beamwidth of aprox. 45° in elevation and azimuth. They have been selected for both transmitting and receiving antennas because of their reduced sidelobes in the endfire direction (Figure 5). The array can operate in both vertical and horizontal polarizations. The use of these planar structures, with acceptable antenna efficiency and reduced dimensions and weight, facilitate their integration in the lateral wall of the aircraft fuselage without affecting its aerodynamics. Since small antennas are used, the resulting low gain is sufficient for short range land/sea observation. In addition, the wide antenna beamwidth together with the Polarimetric and Interferometric capabilities require a high Pulse Repetition Frequency (PRF) to avoid aliasing in the along-track axis.

The whole payload of every prototype (RF unit, digitizer, computer and batteries) weights 5 kg and its dimensions are 300 × 170 × 200 mm^3^. The overall power consumption, including RF and Control Unit, is less than 75 W.

Special care must be taken in the use of DC/DC switching power regulators. The electrical noise generated by these circuits can affect notably the operation of the system resulting in possible image artifacts if not properly filtered.

A second generation of the X-band unit is under test, where the dimensions have been reduced to 250 × 170 × 140 mm^3^ (it includes the RF unit, the computer and the digitizer board) with a weight of 2.5 Kg and a power consumption of 50 W.

**Figure 6.**
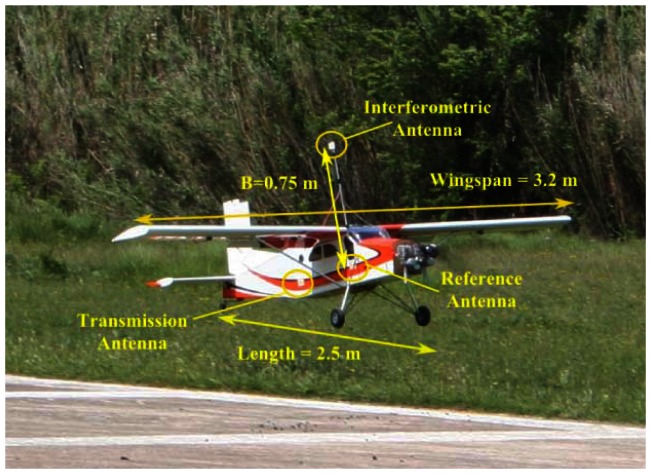
UAV with ARBRES-X for single-pass interferometric SAR measurements. Two antennas (transmitter and one receiver) are attached to the fuselage. The antenna of the interferometric channel is located on top of a metallic support (baseline, B = 0.75 m).

The reduced dimensions and low weight of the instruments allow the use of common Radio Control model airplane as the aerial platform, in this case a scale model of the commercial Pilatus Porter (Pilatus Aircraft Ltd). The platform specifications can be found in Table 2. Figure 6 is a picture of the UAV showing the setup of the antennas for single-pass interferometric SAR measurements in the X-band.

## 4. Signal Processing

Different SAR processing techniques can be used to focus the acquired raw data in stripmap imaging, *i.e.*, the Range-Doppler, omega-K (ω-k), the Chirp Scaling and Back Projection (BPA) [11,12] algorithms. All these algorithms assume that the platform follows a straight trajectory with constant velocity and height. Due to flight instabilities, airborne SAR data is often acquired along nonlinear trajectories. The actual platform track, together with attitude and velocity fluctuations must be measured and compensated for in the SAR processing [13]. In the case of small UAV-SAR the amount of acquired data is small. Accordingly, for preliminary data quality assessment, a BPA has been used despite its high computational cost. The BPA offers a high degree of flexibility in focusing extended images from arbitrary synthetic apertures limited either by antenna beam, linear track or data acquisition capacity [14,15]. In addition Motion Compensation can easily be included even in the case of large flight instabilities [13]. After range compression, the BPA reconstructs the image pixels by adding coherently the back propagated data acquired along the synthetic aperture. Under the stop-and-go assumption (the radar platform is assumed stationary during the transmission of the electromagnetic pulses and the reception of the corresponding echoes), the BPA can be analyzed in a simplified way as follows:

Considering that the transmitted signal is an ideal Linear Frequency Modulated CW chirp:

(1) $$s_{T}(t)=a(t)\cdot e^{j(\beta t+\alpha t^{2})}$$

where *a*(*t*) is a rectangular function corresponding to a chirp period, β is the carrier frequency and α the chirp rate. For a certain antenna position *Ap_i_* with spatial coordinates (*x_ai_*, *y_ai_*, *z_ai_*), the received signal, after the analog deramping will be [11]:

(2) $$s_{Ri}(t)=\sum_{k}B\cdot a(t-\tau_{k})\cdot \sigma_{k}\cdot e^{j(\beta \tau_{k}-\alpha {\tau_{k}}^{2})}\cdot e^{j(2\alpha \tau_{k})\cdot t}$$

where *τ_k_* is the round trip delay of the echo from target *k* (with a radar cross section *σ_k_*) to the antenna position *Ap_i_*. Since each scene scattering center will produce a beat frequency 2*ατ_k_* proportional to the scatterer delay, range compression can be obtained with a Fourier Transform of Equation (2) as follows:

(3) $$S_{Ri}(\omega )=\sum_{k}B\sigma_{k}\;e^{-j(\beta \tau_{k}+\alpha {\tau_{k}}^{2}+\omega \tau_{k})}\cdot A(\omega +2\alpha \tau_{k})$$

where *A*(*ω*) is the Fourier transform of *a*(*t*) and *aτ_k_*^2^ + *ωτ_k_* is the *residual video phase* error. This last term must be cancelled for a correct cross-range image reconstruction. The constant *B* includes the transmitted power, antenna and receiver power gain and propagation losses. Each pixel of the SAR image *p(x,y,z)* is reconstructed by the coherent sum of the back-propagated contribution of the received signal at every antenna position along the synthetic aperture. This sum can be written as [12]:

(4) $$p(x,y,z)=\sum_{i=1}^{n}S_{Ri}\left(2\alpha \tau_{i}\right)\cdot e^{j\;\beta \tau_{i}}$$

where *τ_k_* is the round trip delay of the echo from the evaluated pixel at (x,y,z) to the antenna position *Ap_i_* (*x_ai_*, *y_ai_*, *z_ai_*):

(5) $$\tau_{i}=\frac{2\cdot \sqrt{{\left(x-x_{ai}\right)}^{2}+{\left(y-y_{ai}\right)}^{2}+{\left(z-z_{ai}\right)}^{2}}}{c}$$

Expression (5) shows that any error in the estimation of the antenna position (*x_ai_*, *y_ai_*, *z_ai_*), will affect the focusing process (4). Note that the back-propagation factor in Equation (4) is simply the 2-way phase lost in the observation path. No amplitude compensation is used because the receiver includes a Sensitivity Time Control (STC) subsystem in order to maintain a constant receiver backscattering sensitivity with range. The STC has been implemented with a base-band filter after deramping with the appropriate high-pass compensation law. The upper cut-off frequency of this filter limits the swath to 3 km in both sensors. For simplicity the previous formulation does not take into account the weighting factor of the SAR antennas pattern that should be included for a correct radiometric compensation.

The stop-and-go approximation is valid for both C and X band versions of the SAR sensor. For larger modulation periods or faster platforms the stop-and-go approximation will eventually breakdown resulting in spreading and shifting at the range compression step [16]. However, this degradation can be avoided with the inclusion of compensation techniques in the BPA as shown in [17,18]. Using the IMU-GPS data, synchronized with the radar acquisition [19], it is possible to improve the accuracy of the pixel-antenna delays *τ_i_* in Equation (5), compensating in this way the motion induced errors in the SAR processing.

## 5. Experimental Results

**Figure 7.**
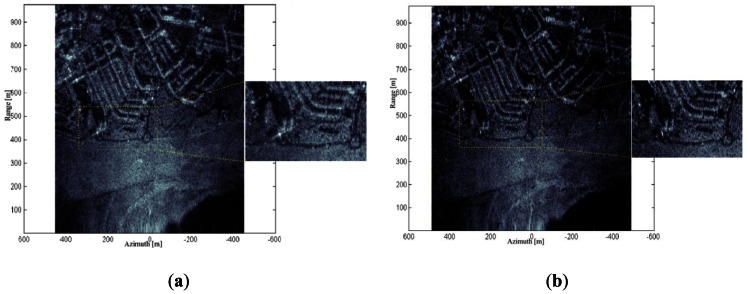
C-band SLCs, (**a**) without and (**b**) with MoCo. The slant range is on the vertical axis. Flight conditions: 200 m height, v_uav_ = 37 m/s, 40 m aperture length. VV Pol.

Different measurement campaigns have been done in order to validate the correct performance of the two systems, all of them at Real AeroClub Barcelona-Sabadell (RACBSA) Radio Control Airfield (Ripollet, Barcelona, Spain). The scene contains agricultural fields surrounded by an urban area with a smooth topography. Figure 7(a,b) shows two Single Look Complex (SLC) images in the C-band obtained from the same raw data. The first one has been generated assuming an ideal trajectory and, as expected, the image presents some degree of defocusing. The second one has been reconstructed using the aircraft trajectory retrieved from the IMU-GPS unit when processing the raw data with the BPA. As foreseen the motion compensation results in better focused images, with higher contrast and spatial resolution.

In these preliminary experiments the synthetic aperture length has been limited by the data acquisition subsystem instead of the antenna beamwidth, due to the available memory depth of the digitizer that limits the maximum raw data recording length. Accordingly the resulting azimuth resolution is worse than the SAR theoretical limit depending on both distance and wavelength.

**Figure 8.**
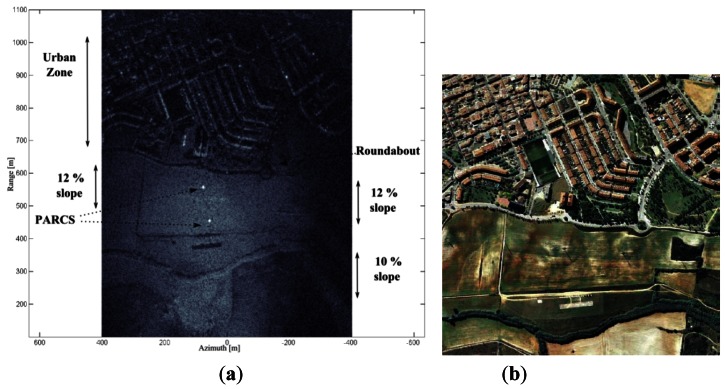
(**a**) X-band SLC SAR image obtained with MoCo. The increase of spatial resolution is noticeable compared with the C-band image of Figure 7. Flight conditions: 300 m height, v_uav_= 37.35 m/s, 120 m aperture length. VV Pol. (**b**) *Google-Earth* image of the scenario.

Another SLC image, this time in the X-band, is shown in Figure 8. The resolution is better in both dimensions, as the bandwidth is twice that of the C-band instrument and the wavelength is shorter. It is easy to distinguish strong scatterers located at buildings, roundabouts and the polarimetric active radar calibrators (PARCs) deployed as ground control points. These PARCs were deployed to characterize the SAR spatial resolution and the minimum detectable backscattering.

**Figure 9.**
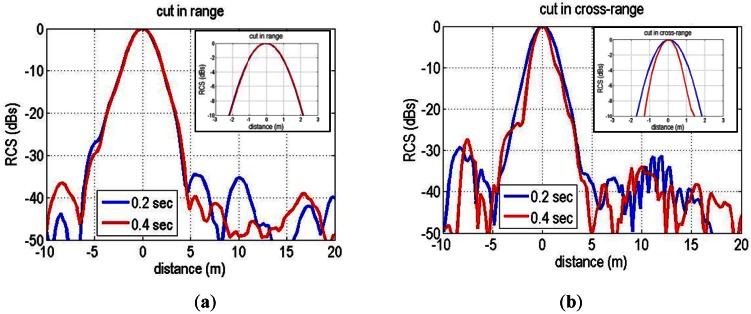
X-band pixel response characterization of the PARC under study. (**a**) normalized cut in range for the two different aperture lengths. (**b**) normalized cut in cross-range.

Figure 9 shows the range and cross-range cuts of the normalized Radar Cross Section (RCS) of the closer PARC for two different aperture length (0.2 and 0.4 s, that correspond to approximately 7.5 and 15 m respectively). With these short apertures, the cross-range resolution is range dependent and can be calculated by means of the −3 dB synthetic antenna beamwidth estimated to be 0.2° (7.5 m) and 0.1° (15 m), multiplied by the pixel slant range (600 m). The obtained range resolution in the order of 2 m is slightly above the nominal value ΔR = c/2B = 1.5 m. This resolution broadening is caused by the application of a tapering hanning window on the analog deramped signal for sidelobe control in the range compression process. The cross range resolution is in good agreement with the theoretical value in the case of the shortest aperture (7.5 m). In the longer aperture case the cross range resolution is below the theoretical prediction, which can be associated to different sources: the use of tapering window functions in the cross-range focus processing, the effect of the squint angle of the target and a non-perfect motion compensation of the platform (more accentuated in the longest aperture). Nevertheless, these measurements show that the use short aperture lengths are sufficient for reaching a reasonable range/cross-range resolution for images limited to several kilometers in length.

The sensitivity of a SAR system is usually expressed with the Noise Equivalent Sigma Zero (NESZ) parameter, defined as the surface backscattering coefficient resulting in equal signal and noise powers in the SAR image. Comparing the PARCs imaged amplitude with their known RCS the image has been calibrated. On the other hand the image noise power has been estimated from the near range band of the image where no echoes are expected. In this way a NESZ value of −32 dB has been obtained, which is in good agreement with the theoretical calculated value [11].

**Figure 10.**
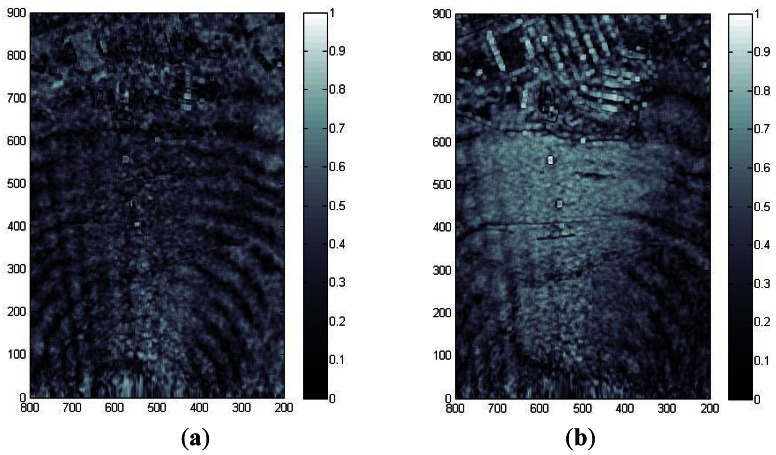
Coherence map from a 100 MHz bandwidth, single-pass, interferometric X-band measurement. (**a**) without and (**b**) with MoCo (x-axis is azimuth and y-axis range in meters).

**Figure 11.**
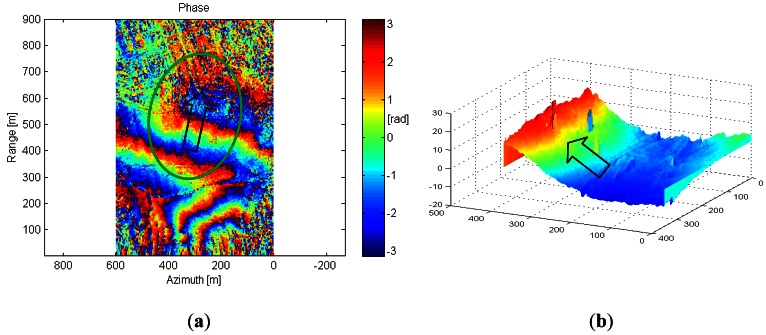
(**a**) Phase map (in rad) from an interferometric measurement in single-pass mode over the same scenario. (**b**) Extracted Digital Elevation Model (DEM) of part of the scenario after phase unwraping and geocoding (Peaks correspond to PARCs and power line towers). The ellipse shows the zone of interest and the arrow the orientation of the slope where the DEM is retrieved.

In order to generate the single pass interferometric measurements, the X-band ARBRES system uses a second receiving antenna placed with a vertical baseline of 75 cm above the reference fuselage antenna. Figure 10 shows the coherence map, where the values are above 0.8 in most areas of the image. Figure 11(a) shows the phase interferogram after flat earth removal and aircraft attitude compensation and Figure 11(b) is the equivalent Digital Elevation Map (DEM) obtained from the interferogram after phase unwraping and geocoding. In this case the synthetic aperture length was 80 m.

## 6. Conclusions

In the ARBRES project, C band and X band highly compact dual channel SAR systems conceived for small UAVs have been developed and validated. The sensors are suited for testing innovative multichannel architectures and techniques requiring direct access to raw-data. Both sensors have two receiver chains to carry out single-pass interferometric or polarimetric measurements. The reduced dimensions, weight (10.2 dm^3^ and 5 Kg) and power consumption (less than 75 W) of the sensors and controller allow the use of a 3m wingspan radio controlled model airplane as the aerial platform.

A Back-Projection algorithm has been used to focus the acquired raw data. Defocusing and aberration in the SLC image formation due to non-ideal flying path of the UAV has been corrected via Motion Compensation, using the position and attitude information of the platform measured with an INS+GPS unit. Results showed that reasonable resolution values (better than 2 × 2 m^2^) are obtained in small images (several km^2^), even in the case of operating the radar with short aperture lengths. In this way commonly available electronics for data acquisition and relative small memory banks can be used. A single pass interferogram has been obtained at X-band which shows the correct performance of the dual channel architecture. Further research will be focused on polarimetric, interferometric and Moving Target Indication techniques applications from UAV platforms.

## Figures and Tables

**Table 1.** ARBRES specifications.

**Parameter**	**C-Band**	**X-band**
Central frequency	5.3 GHz	9.65 GHz
Bandwidth	50 MHz	100 MHz
PRF	20 kHz	
Pout	30 dBm	
Receiver chain Gain	up to 70 dB	
Range resolution (nominal)	∼3 m	∼1.5 m
Theoretical Swath	7.5 km	3.75 km

**Table 2.** UAV specifications.

**Wingspan**	3.2 m
**Length**	2.5 m
**Propulsion**	100 cc Bicylinder gasoline engine
**Cruise Speed**	35 m/s
**Takeoff Weight**	17 kg (with sensor)
**Power Supply**	14.8 V 3,600 mAh LiPo Batt
**Hold Dimensions**	400 × 280 × 240 mm^3^ (l × h × w)
**Cruise Speed**	40 m/s
**Flight Altitude**	Limited by local regulations. Less than 300 m
